# The use of internet platforms for oral health information and associated factors among adolescents from Jakarta: a cross sectional study

**DOI:** 10.1186/s12903-020-01387-x

**Published:** 2021-01-07

**Authors:** Diah Ayu Maharani, Maha El Tantawi, Marsha Griselda Yoseph, Anton Rahardjo

**Affiliations:** 1 Department of Preventive and Public Health Dentistry, Faculty of Dentistry, Universitas Indonesia, Jalan Salemba No. 4, Jakarta, 10430 Indonesia; 2 Department of Pediatric Dentistry and Dental Public Health, Faculty of Dentistry, Alexandria University, Alexandria, Egypt

**Keywords:** Adolescents, Oral health information, Social media, Indonesia

## Abstract

**Background:**

The growth of the internet has increased its use to obtain health information including oral health information (OHI). This study assessed Indonesian adolescents’ use of different internet platforms to obtain OHI and factors associated with this use.

**Methods:**

A cross-sectional study surveyed middle school students in five regions in Jakarta in 2019. Participants completed a questionnaire that assessed demographics, oral health practices (toothbrushing and dental visits), the presence of dental pain, using internet platform to obtain OHI and type of information searched for. Multinomial logistic regression was used to assess the association between using the internet for OHI (Google, Social Media (SM), both or none) and the independent factors: demographics, oral health practice, dental pain and whether participants search for causes, symptoms, prevention or treatment of oral diseases (ODs).

**Results:**

Most of the 521 participants were female (55.7%) with mean age = 13.4 years. Almost all of them (93.7%) searched the internet for OHI through Google (40.7%) or Google with SM (36.1%). Searching for OHI over SM was significantly associated with toothbrushing (OR = 4.12, 95% CI = 1.43, 11.89) and less dental visits (OR = 0.16, 95% CI = 0.05, 0.60). Searching Google for OHI was significantly associated with looking for information about causes (OR = 3.69, 95% CI = 1.33, 10.26) and treatment (OR = 6.17, 95% CI = 2.23, 17.03) of ODs.

**Conclusions:**

Most adolescents used Google to seek OHI. Oral health practices and types of OHI searched for differed by internet platform. Dental health professionals should consider using internet-based interventions to promote oral health to this age group.

## Background

Adolescents usually have high prevalence of dental caries, trauma, and periodontal disease [1]. A previous study reported a high prevalence of oral diseases among Indonesian adolescents where 61% of 12-year-old adolescents in Jakarta, Indonesia had dental caries [2]. Neglecting oral hygiene in adolescents negatively affects dental caries, periodontal diseases in addition to social and emotional well-being [3]. Oral hygiene may also have implications on social acceptability and self-esteem [4].

When children are young, decisions are predominantly made by parents or caregivers because children’s decision-making abilities are not adequately formed. During adolescence, cognitive functioning improves with age and teenagers can start making and applying decisions to obtain the best possible outcome [5]. Four capacities are required for adolescents to make their own healthcare decisions: expressing a choice, understanding, reasoning, and appreciation [6]. At this developmental stage, behavior modification is important to foster proper self-care habits that can help reduce oral diseases and hopefully last throughout life [7].

Using social media (SM) is one of the most common activities of today's adolescents. Websites that allow social interaction are considered SM platforms, allowing users to communicate, develop their creativity, expand their knowledge and obtain health information. Searching for health information online may make users feel more secure in expressing their primary concerns because their identities are masked and their privacy is guaranteed [8] and users can engage with content generated by others [9]. Health information can be disseminated through a variety of forms over SM such as blogs, podcasts, tweets, Facebook pages or posts, and YouTube videos [10]. A previous study showed that adolescents also used SM to seek oral health information (OHI) [11]. This may be especially relevant in Indonesia where it was reported that users spend more time on the internet than users in other parts of the world [12]. Thus, the internet may offer an opportunity to disseminate OHI targeting Indonesians adolescents.

Understanding how OHI is obtained over the internet helps in developing internet-based health education programs and allows the selection of the proper internet platform to disseminate OHI for adolescents so that health education messages are tailored to the needs and interests of the target group with potentially better engagement. It is important to understand whether adolescents use SM to search for OHI thus depending on word of mouth and experiences of peers or search Google which may direct them to websites of professional organizations, dentists or companies selling oral health care products. The objectives of this study were to assess the use of different internet platforms to obtain OHI by adolescents in Jakarta, and the factors associated with this use. The null hypothesis of the study was that adolescents would equally use search engines such as Google and SM to obtain OHI.

## Methods

### Study design and participants

A cross-sectional, questionnaire-based study was conducted among middle school students in five areas of Jakarta (North, South, East, West, Central) in October 2019. Jakarta is the capital and largest city of Indonesia which is the 4th most populous country in the world [2]. Sample size was calculated based on assuming a margin of error = 5%, confidence level = 95%, and percentage of adolescents using SM to obtain OHI = 58% [11]. The required sample size was 374 participants, increased by 30% to compensate for potential nonresponse. Adolescents were included if they went to middle school (private or public) in Jakarta, had no physical or intellectual disability preventing them from responding to the questionnaire and if their parent/guardian consented to their participation and they assented. The government provided a list of middle schools in the city. In each of the five administrative districts of Jakarta, schools were randomly selected so that it would be possible to recruit a number of students proportional to the size of the population living in that district. Participants were selected using cluster sampling from each school. This method resulted in the random selection of a total of 14 schools with 50 students from Central Jakarta, 70 students from North Jakarta, 148 students from West Jakarta, 109 students from South Jakarta and 144 students from East Jakarta.

### Data collection tool

A questionnaire was designed based on the one used by El Tantawi et al. [11]. The original questionnaire was translated to Bahasa Indonesia, the main language used in Indonesia then back translated to English and compared with the original English version to resolve inconsistencies. Some words were modified so that their meaning in the Bahasa Indonesian version would be similar to the original English version. Further, a pilot test of the modified questionnaire was conducted among adolescents to determine clarity and comprehensiveness of the wording. The final questionnaire was used as a self-administered Bahasa Indonesia form. The questionnaire was divided into three sections. The first section collected personal information including gender, date of birth, whether parents were university educated and whether the mother was a housewife. The second section assessed oral health practices including brushing twice daily and regular visits to the dentist and presence of dental pain in the last six months using close-ended questions with yes or no responses. The third section assessed the use of Internet for OHI. Participants were asked about the internet platforms they used to search for OHI: whether they used Google or a number of SM. They were also asked about the OHI they looked for covering aspects of oral diseases (ODs) including causes, symptoms, prevention and treatment. The students received the questionnaires on school premises and were given 5–10 min to respond to its items anonymously and the forms were collected afterwards. Data were entered into IBM SPSS for Windows version 23.0 (IBM Corp., Armonk, N.Y., USA) and cleaned for analysis.

### Data analysis

Descriptive analysis was conducted and summary measures were calculated as means and standard deviations or frequencies and percentages. A new variable “using SM” was created by counting users of any type of SM. The dependent variable was using various internet platforms to obtain OHI (categorized into using Google, SM, both, or none) and the independent variables included factors reflecting interest in oral health based on their adoption of oral health practices such as brushing and dental visits and the need for OHI due to the presence of dental pain. The independent factors also included searching for OHI about various aspects of ODs. Multinomial logistic regression analysis was used to assess the association between the dependent and independent variables adjusted for personal factors as confounders. Odds ratios and 95% confidence intervals (CIs) were calculated. Significance was set at 5%.

## Results

The parents of all students who were invited to join the study consented to their participation and the students assented (n = 521, response rate = 100%). Table 1 showed that most participants were female (n = 290; 55.7%) with mean age = 13.4 ± 1 year old. The age of the respondents ranged from 11–17 years old. Most parents were non-university educated (father = 65.3% and mother = 67.4%), with half mothers being housewives (49.1%). Most participants brushed twice daily (81.4%), did not regularly visit the dentist (88.3%) and had no dental pain in the last 6 months (77.9%). The overall average time that they spend using the internet per day is 3.2 ± 1.9 h per day.

**Table 1:** Personal profile, toothbrushing, dental visits and dental pain among students participating in the study

Variables	n (%)
Gender	
Male	231 (44.3)
Female	290 (55.7)
Father’s education	
University educated	181 (34.7)
Non-university educated	340 (65.3)
Mother’s education	
University educated	170 (32.6)
Non-university educated	351 (67.4)
Housewife mother	
Yes	256 (49.1)
No	265 (50.9)
Brushes twice daily	
Yes	424 (81.4)
No	97 (18.6)
Regular visit to the dentist	
Yes	61 (11.7)
No	460 (88.3)
Had dental pain in the last 6 months	
Yes	115 (22.1)
No	406 (77.9)

The results show that 40.7% used Google to search for OHI, 16.9% used SM and 36.1% used both Google and SM with a total of 93.7% using some internet platform to search for OHI. Participants using SM used YouTube (40.7%), Instagram (24%), Facebook (5%) and Twitter (1.7%). About 49.3% reported searching for OHI about treatment, 47.6% about prevention, 43.4% about causes and 14.6% about symptoms of ODs.

Table 2 shows that various factors were differently associated with using Google, SM or both compared to not using the internet at all to obtain OHI. Brushing twice daily was associated with significantly greater likelihood of using SM (OR = 4.12, 95% CI = 1.43, 11.89) and both Google and SM (OR = 2.70, 95% CI = 1.03, 7.08) but was not significantly associated with using Google alone (OR = 2.10, 95% CI = 0.84, 5.24). Regular dental visits were associated with significantly lower likelihood of using SM (OR = 0.16, 95% CI = 0.05, 0.60) but had no significant association with using Google alone (OR = 0.42, 95% CI = 0.15, 1.17) or using both Google and SM (OR = 0.42, 95% CI = 0.14, 1.23). Exclusive users of Google and SM alone were significantly more likely than those who did not use the internet for OHI to be looking for information about ODs causes (OR = 3.69, 95% CI = 1.33, 10.26 and 3.49, 95% CI = 1.18, 10.26) and treatment (OR = 6.17, 95% CI = 2.23, 17.03 and OR = 5.13, 95% CI = 1.75, 15.01) while users of both Google and SM together were significantly more likely than non-users of the internet to be looking for OHI about symptoms (OR = 6.07, 95% CI = 1.24, 29.87) and prevention (OR = 4.82, 95% CI = 1.95, 11.89) in addition to causes (OR = 6.98, 95% CI = 2.46, 19.79) and treatment (OR = 9.86, 95% CI = 3.46, 28.06).

**Table 2:** Factors associated with using Google, SM, and Google and SM combined for OHI versus not using the internet for OHI by adolescents in Jakarta, Indonesia

	OR (95% CI)		
	Google	SM	Google and SM combined
Brushes twice daily	2.10 (0.84, 5.24)	4.12 (1.43, 11.89)*	2.70 (1.03, 7.08)*
Visiting the dentist regularly	0.42 (0.15, 1.17)	0.16 (0.05, 0.60)*	0.42 (0.14, 1.23)
Had dental pain in the last 6 months	0.92 (0.36, 2.33)	1.36 (0.50, 3.69)	1.12 (0.43, 2.96)
Searching for ODs causes	3.69 (1.33, 10.26)*	3.49 (1.18, 10.26)*	6.98 (2.46, 19.79)*
Searching for ODs symptoms	2.26 (0.46, 11.06)	1.58 (0.28, 8.88)	6.07 (1.24, 29.87)*
Searching for ODs treatment	6.17 (2.23, 17.03)*	5.13 (1.75, 15.01)*	9.86 (3.46, 28.06)*
Searching for ODs prevention	2.22 (0.93, 5.32)	1.66 (0.65, 4.25)	4.82 (1.95, 11.89)*

Adjusted for region, gender, age, parents’ education and mother’s job

^*^Statistically significant at P < 0.05

## Discussion

The study showed that the internet was used by almost all the adolescents participating in the study to obtain OHI with the majority using Google alone or in combination with SM. The search mainly focused on treatment and causes of ODs. Using SM for OHI was less likely for those who visited the dentist regularly and those who did not brush their teeth. The findings highlight the importance of the internet as a source of OHI in this vulnerable age group and draw the attention of dental professionals to the need to monitor the accuracy of OHI which is posted in most cases without quality control [13]. The findings also suggest the possibility of using the internet to offer OHI to adolescents using websites of professional organizations and other agencies that can be retrieved through Google search. The evidence does not support the claim that the SM are the most frequent source of OHI for adolescents and thus, the null hypothesis of the study is rejected. The study showed the ubiquitous use of internet and SM by adolescents. This agrees with previous studies that highlighted the ease of use and accessibility of the internet and SM through mobile devices where they can be accessed and used any time [11, 14].

Google was the most popular internet platform for OHI in the present study. This agrees with another study where the role of Google as a search engine was reported to be particularly important because Google actively mediates and shapes the information seen by its users [14]. Previous research showed that search engines such as Google offered a variety of content and had minimal advertisements. Information provided through Google can be checked through its preview feature without the need to visit the original website, thus allowing users to skim through information directly [15]. The present findings agree with Fan et al. [16] who reported that adult users searched Google more than SM for information about plastic surgery. Google have shown how the pervasive internet, connectivity, big data analytics and artificial intelligence can be used to dissolve boundaries and constraints, build closer relationships with adolescents, learn more about their behaviors and preferences, and deliver highly personalized experiences and products in sustainable and cost-effective ways [17].

The present study showed that YouTube was the most popular SM for OHI. The videos offered on YouTube engage multiple senses, including hearing and seeing at the same time which is helpful for information retention. People may remember up to 10% of what they read, 20% of what they hear, 30% of what they see, and 50% of what they see and hear [18]. The study findings differ from those of El Tantawi et al. where Saudi adolescents preferred using Instagram for OHI [11] indicating that use of different types of SM for OHI may differ by country or culture or even by time as certain SM become popular. For example, a 2012 survey [19] showed that Jakarta was the most active Twitter city in the world although the use of Twitter in the present study was reported by a minor portion of users. The difference between that report and the present findings may be explained by the eight years that passed since this report during which the SM stage in the country has changed. By contrast, another recent report in 2019 [20] showed that YouTube was the most popular SM in Indonesia with penetration rate > 88% which agrees with the predominance of YouTube observed in the current study.

The study showed that using SM for OHI was directly associated with toothbrushing. This may suggest that searching for OHI over SM is more frequent among those who already adopt self-care practices such as tooth brushing to maintain oral health. The findings also showed that searching SM for OHI was inversely associated with regular dental visits. This may be attributed to the availability of OHI through the dentist during these visits which reduces the need for searching for answers to questions about ODs over SM.

In the present study, dental pain was directly associate with obtaining OHI from SM and inversely associated with obtaining OHI from Google. This agrees with Bounsanga et al. [21] who reported better health among adults with health information obtained from the internet but not with information obtained from SM. The associations observed in the present study were not statistically significant possibly indicating minimal impact of OHI on oral health status and dental pain which may be partly explained by the low prevalence of dental pain in the present study reducing the power to detect significant associations in addition to the young age of the participants which allowed limited duration for OHI to affect oral health status and pain. However, it is important to interpret these findings within the framework of the cross-sectional nature of the study which makes it impossible to prove that participants were exposed to OHI before pain assessment and that OHI may, therefore, potentially reduce/change pain. Further longitudinal studies are needed to assess causality between exposure to OHI from various sources and oral status or dental pain. Confounder identification is important. Personal factors were considered as confounders in this study. Without proper adjustment for confounders, the association will be a biased estimate of the true association. The confounders were adjusted by controlling it with appropriate statistical techniques [22].

The present study draws attention to the importance of OHI on the internet because of the great interest of adolescents in it. Dental professionals may need to direct their attention toward these tools for the dissemination of OHI. Translating research findings into easy-to-understand language and disseminating these findings to attract the attention of online users may help provide evidence-based OHI material [23]. There is a need to explore the quality of OHI currently available on Google and YouTube in Bahasa Indonesia. The present study is limited by its cross-sectional design which suggests association but cannot prove causality. The study included adolescents only from Jakarta which is one city in Indonesia. However, it is the largest city in the country and the profile of its population generally reflects the characteristics of the population including adolescents all over the country [2].

Adolescents possessing capacities required for decision-making, such as understanding the choices and reasoning of the decision being made, may need support of facilitating environmental factors [6]. This process might be explained by behavioral theories. The social learning theory assumes that behavior is learnt from the environment through observation [24]. This suggests that SM imagery content has the potential to influence oral health behavior. Furthermore, the theory of presumed media influence persuasive media messages influence attitudes indirectly by changing perceptions of descriptive norms among peers [25]. Another theory, the theory of planned behavior [26] treats attitudes and norms as separate factors influencing behavior and does not consider how beliefs and attitudes might influence injunctive norms. Therefore, it is essential to further conduct studies to develop appropriate OHI for adolescents, given the possibilities of different theories as a base of explaining the efficacy of the information delivered. SM has the potential to reach and influence a broad audience, particularly as a means of engagement rather than just disseminating information. Audio-visual SM may be more efficient for oral health promotion amongst adolescent when compared to solely text-based medium [27]. Clinicians interested in using YouTube and other SM for broad reach to adolescents may benefit from engaging adolescents in creating powerful and effective SM messages. It was reported that adolescents were more engaged with videos posted by their peers, which may have been viewed as easier to understand [28].

## Conclusion

Based on the findings of this study, most adolescents in Jakarta used the internet to obtain OHI focusing more on Google than SM. There are differences among adolescents searching Google and SM for OHI based on oral health practices and the type of OHI addressing specific aspects of ODs.

## Data Availability

The raw data are available from the authors to any author who wishes to collaborate with us.

## Abbreviations

OHI: Oral health information
SM: Social media
ODs: Oral diseases
